# Authors’ Response to Peer Reviews of “Improved Alzheimer Disease Diagnosis With a Machine Learning Approach and Neuroimaging: Case Study Development”

**DOI:** 10.2196/72821

**Published:** 2025-04-21

**Authors:** Lilia Lazli

**Affiliations:** 1 Department of Computer and Software Engineering, Polytechnique Montréal, University of Montreal, 2500 Chem de PolytechniqueMontreal, QC, H3T 1J4, Canada, 1(514) 340-5121 ext 3750

**Keywords:** Alzheimer disease, computer-aided diagnosis system, machine learning, principal component analysis, linear discriminant analysis, t-distributed stochastic neighbor embedding, feedforward neural network, vision transformer architecture, support vector machines, magnetic resonance imaging, positron emission tomography imaging, Open Access Series of Imaging Studies, Alzheimer's Disease Neuroimaging Initiative, OASIS, ADNI

*This is the authors’ response to peer-review reports for “Improved Alzheimer Disease Diagnosis With a Machine Learning Approach and Neuroimaging: Case Study Development.”*

## Round 1 Review

### Anonymous [1]

#### General Comments

*This paper [2] proposes a computer-aided diagnosis (CAD) system for Alzheimer disease (AD) using principal component analysis (PCA) and machine learning–based approaches. The authors claim that their system, which combines PCA for feature extraction with support vector machines (SVMs) and artificial neural networks (ANNs) for classification, achieves good accuracy in detecting AD from magnetic resonance imaging (MRI) and positron emission tomography (PET) images. However, the paper could be strengthened by addressing several areas for improvement.*

#### Specific Comments

##### Major Comments

*1. Consideration of alternative methodologies: While the use of PCA, SVMs, and ANNs for AD classification is a valid approach, the authors should consider exploring more recent deep learning architectures, such as vision transformers (ViTs), which have demonstrated state-of-the-art performance in medical image analysis. This would help to situate the work within the broader context of current research in the field.*

**Response:** Done, please see the Transformers subsection (page 5). The results obtained and the discussion on the potential of this approach are mentioned in the Results (page 7) and Discussion (page 8) sections, respectively. Moreover, details on the mathematical background can be found in Multimedia Appendix 4: Vision transformer.

*2. Limited evaluation: The evaluation is limited to the Open Access Series of Imaging Studies (OASIS) dataset, which may not be representative of the diverse AD population. The authors should evaluate their system on larger and more diverse datasets, such as the Alzheimer’s Disease Neuroimaging Initiative (ADNI) dataset, to demonstrate its generalizability.*

**Response:** Done, experiments were achieved by applying the ADNI database. Please see the ADNI Data Set subsection (page 3) for more details on this basis. Table 1 (page 6) and Table 2 (page 7) for demographic characteristics and clinical assessments as well as the Results (page 7) and Discussion (page 8) sections.

##### Minor Comments

*1. Insufficient implementation details: The implementation details of the SVMs and ANNs are insufficient. The authors should specify the hyperparameters used, such as the kernel type and regularization parameters for SVMs, and the number of layers and neurons for ANNs.*

**Response:** Done, please see Table 3 (page 7).

*2. Limited discussion: The discussion of the results is limited. The authors should provide a more in-depth analysis of the performance of their system, comparing it with other state-of-the-art methods and discussing the limitations and potential future directions.*

**Response:** Done, please see the Discussion section (page 8).

*3. The authors should ensure consistent formatting throughout the paper, including the use of italics for variables and proper capitalization in section headings.*

**Response:** Done, the format of the journal was generally respected.

*4. The paper could be improved by using more precise language. For instance, instead of “good accuracy,” the authors could specify the exact accuracy percentage achieved by their system.*

**Response:** Done, precisions for the decimal values of the results obtained are mentioned in the Results and Discussion sections, and in the abstract.

### Reviewer AS [3]

#### General Comments

*This paper explores the use of PCA and machine learning approaches for the diagnosis of AD using MRI and PET images from the OASIS database. The authors propose a system that combines PCA for feature extraction with ANNs and SVMs for classification. The paper is well structured and presents a clear methodology, but there are several areas where improvements are needed to enhance the rigor and impact of the research.*

#### Specific Comments

##### Major Comments

*1. Methodology justification: The choice of PCA as the sole feature extraction method needs further justification. While PCA effectively reduces dimensionality, it might not capture the most discriminative features of AD. Comparing PCA with other dimensionality reduction techniques like linear discriminant analysis or t-distributed stochastic neighbor emulation could provide a more comprehensive understanding of its effectiveness.*

**Response:** Done, a comparative study was performed between these three dimensionality reduction techniques. Please see Table 4 and Table 5 (page 9) for the results and the Discussion section (page 8, especially lines 29-36).

*2. Evaluation metrics: The paper primarily focuses on accuracy as the evaluation metric. For medical diagnosis systems, metrics like sensitivity, specificity, precision, recall, and F_1_-score are crucial as they provide a better understanding of the model’s performance, especially in imbalanced datasets. Including these metrics would strengthen the evaluation section.*

**Response:** Done, please see the Statistical Analysis subsection (page 5) and Tables 4 and 5 for the results.

*3. Dataset and preprocessing: The preprocessing steps are briefly mentioned but lack detailed explanation. Specific steps for noise reduction, intensity normalization, and any augmentation techniques used should be clearly described. Additionally, the impact of these preprocessing steps on the model’s performance should be discussed.*

**Response:** Done, please see the Data Preparation section (page 3) and the Discussion section (page 8), particularly, the paragraphs in lines 21 and 22 and lines 45-48.

*4. Comparison with existing methods: The paper lacks a thorough comparison with existing state-of-the-art methods. Including a detailed comparison with recent literature, both in terms of methodology and performance, would provide better context and highlight the novelty and effectiveness of the proposed approach.*

**Response:** Done, please see the Comparison With Prior Work subsection (page 9) and Table 6 (page 10).

##### Minor Comments

*1. Introduction section: The Introduction provides a good overview of AD and the need for early diagnosis. However, it could benefit from a more detailed discussion of the current challenges in AD diagnosis and how the proposed method aims to address these challenges.*

**Response:** The content of the Introduction has been improved to take some challenges into consideration. Please see particularly the paragraph on page 2, lines 34-48.

*2. Figure and table clarity: Figures and tables should be more clearly labeled and described. For example, in Table 1, it is unclear what “Total cost (Validation)” refers to. Additionally, the axes and legends in figures should be more descriptive to enhance readability.*

**Response:** All the content of the paper has been revised and improved by inserting new tables to clearly express the results obtained with the quantitative metrics, suggested by the evaluators. Please see the tables for the detailed results. Furthermore, the results are mentioned in the Results and Discussion sections.

*3. Algorithm parameters: The specific parameters used for the SVMs and ANNs (eg, kernel type for SVMs, number of layers, and neurons for ANNs) should be explicitly mentioned. This would help in reproducing the results and understanding the model configuration.*

**Response:** Done, please see Table 3 (page 7).

*4. Conclusion and future work: The conclusion should be concise and focus on key findings. The Future Work section could be expanded to include more specific directions for further research, such as exploring different feature extraction methods, incorporating longitudinal data, or integrating other imaging modalities.*

**Response:** This section has been deleted and replaced with the Discussion section (page 7) in order to respect the format of the journal. In this section, several subsections were inserted with content responding to your suggestion such as Main Finding (page 8) and Limitations and Future Directions (page 14).

*5. References: Ensure all references are up-to-date and relevant. Given the rapid advancements in machine learning and medical imaging, some references are slightly outdated. Including more recent studies would enhance the credibility and relevance of the paper.*

**Response:** Done, please see the references highlighted in yellow.

### Anonymous [4]

#### General Comments

*The paper discusses the development of a machine learning–based CAD system for the detection and classification of AD. The system uses brain MRI and PET images from the OASIS database, applying PCA for feature extraction and using SVMs and ANNs as classifiers. Although the proposed model shows relatively good performance, the paper should focus on justifying the novelty of the method and providing more details in the results.*

#### Specific Comments

##### Major Comments

*1. The paper lacks a clear discussion on how the proposed method substantially advances the state of the art. While it combines PCA with SVM and ANN, similar combinations have been explored in prior research. The authors should clearly write about how their work is novel and the specific contributions made beyond existing studies.*

**Response:** Please see page 2, lines 34-47.

*2. The paper does not provide sufficient details on the hyperparameter tuning process for both SVM and ANN models. The review suggests that the author include these additional details in an appendix.*

**Response:** Done, Table 3 provides the hyperparameter tuning and classifiers configuration used in the experiment.

*3. The evaluation primarily focuses on accuracy, sensitivity, and specificity. However, other metrics like precision, F_1_-score, and area under the receiver operating characteristic curve could provide a more comprehensive assessment of the model’s performance. The authors could consider adding additional metrics for evaluation.*

**Response:** Done, other metrics were also used. Please see the Statistical Analysis section (page 5) and Table 4 and Table 5 (page 9) for the obtained results.

*4. In Figure 2, the size of the box on the left and right are different (square vs rectangle). Is there a specific reason the author made this design choice?*

**Response:** The figure was removed as more empirical results were inserted responding to the reviewers’ suggestions. Techniques for reducing dimensionality and classification have been added as well as the ADNI database, which has condensed the Results and Discussion sections. I thought it wise to remove certain figures and tables to lighten the paper and avoid redundancy. However, for the design, there is no particular reason. The interface was developed using Matlab toolbox while respecting certain dimensions.

##### Minor Comments

*1. The paper’s organization can be improved. Some sections, like the methodological explanation of PCA, are overly detailed, while others, like the description of SVM and ANN, are relatively brief. Please consider balancing the sections.*

**Response:** Done, all the content of the paper has been revised and improved. Also, appendixes were added to move the entire mathematical background and lighten the paper. Please see the Machine Learning Approaches section (page 3).

*2. The Related Work section is somewhat sparse and does not sufficiently cover recent advances in the field. Please consider adding more recent studies.*

**Response:** Done, please see the Introduction section (page 2), particularly, the paragraph in lines 21-31.

## Round 2 Review

### Anonymous [1]

#### General Comments

*This paper investigates the performance of various machine learning models in the diagnosis of AD using neuroimaging data. The authors propose a CAD system that uses PCA for feature extraction and SVMs, feedforward neural networks, and ViTs for classification. The models are trained and evaluated on two datasets, OASIS and ADNI.*

#### Specific Comments

##### Major Comments

*1. The paper claims that the proposed CAD system is effective in classifying patients with AD and healthy controls (HCs) with high accuracy. However, the reported accuracies of 91.9% for OASIS and 88.6% for ADNI using PCA/SVM are not significantly higher than those achieved by existing state-of-the-art methods (eg, Li Y, Chen G, Wang G, et al. Dominating Alzheimer’s disease diagnosis with deep learning on sMRI and DTI-MD. Front Neurol. Aug 15, 2024;15:1444795. [doi: 10.3389/fneur.2024.1444795] [PMID: 39211812]). A more comprehensive literature review and comparison are needed to support the claim of the proposed system’s superiority.*

**Response:** Performance comparisons between different machine learning techniques by referring to other researchers’ studies are difficult. It is possible that the same algorithm can provide different results for the same database if the study context, the acquisition and learning parameters, the capacity of the computing equipment, etc are different. Nevertheless, to evaluate the effectiveness of the proposed CAD system, a comparative study with some recent works was carried out on the ADNI and OASIS datasets, which we think the development conditions are almost similar to our case.

An objective comparison could not be made with the study proposed in the *Frontiers in Neurology* paper you suggested for two reasons.

1. Researchers used samples from a mixture of two databases, ADNI and Xuanwu Hospital Neuroimaging, to perform the training of the CNN. This provides more data to conduct this process well.
2. Researchers performed two binary classifications (AD vs HCs and mild cognitive impairment [MCI] vs HCs), and they obtained accuracies of 0.96% and 0.83% respectively. In our case, the binary classification performed is AD vs HCs, where samples from patients with MCI and those with confirmed AD are grouped in the same Alzheimer class. The ViT model achieved an accuracy of 90.4% for this category, which is encouraging because MCI is a difficult stage to predict.

*2. The ADNI dataset includes not only patients with AD and HCs but also individuals with MCI. The paper does not explicitly mention whether MCI cases are included in the ADNI dataset used in this study and if patients with MCI are excluded. What is the reason?*

**Response:** Clarifications are provided regarding the subdivision of the two HC and AD classes, which concern HCs and patients with AD, respectively. Please see the related paragraphs on page 3.

*3. The paper’s conclusion that the “PCA/SVM scheme is much better at predicting AD than the other models” is not supported by the results presented. The ViT model with data augmentation consistently outperforms PCA/SVM in terms of accuracy and other metrics. There are no obvious reasons data augmentation is unwanted either.*

**Response:** Details are provided regarding the results obtained with the ViT classifier. Please see the related paragraphs on page 1 and page 2 in the abstract section.

We have confirmed your deduction regarding the performance of the ViT that was applied in conjunction with the data augmentation strategy. We have not criticized the potential of having augmented the data. In general, neural networks in comparison with other machine learning models need a sufficient amount of data to perform their training in order to obtain good results. Therefore, in cases with little data, it is necessary to go through strategies that allow increased data to achieve this objective.

In the paragraph titled Method in the abstract section, we have specified that three classifiers were used: SVM and FFNN with the dimensionality reduction methods as well as ViT with the data augmentation strategy. The Results and Conclusion subsections in the abstract section confirmed that the data augmentation/ViT model outperformed the other models.

##### Minor Comments

*1. The paper claims to use a multimodal system, combining both MRI and PET images. However, it does not compare the multimodal system’s performance against single-modal systems using only MRI or PET images. Such a comparison would help to rationalize the conclusion that the multimodal system truly improves upon single-modal systems.*

**Response:** Please see the related paragraph on page 8.

### Reviewer AS

#### General Comments

*Thank you for addressing my comments from the previous round of reviews. I appreciate the effort you have put into revising the manuscript. The updated version effectively resolves all the issues I raised, and the manuscript is now clear, well-structured, and scientifically sound.*

**Response:** Thank you very much for your valued contribution as well as for your relevant comments in round 1, which helped to improve the contents of the paper.

## Abbreviations

AD: Alzheimer disease
ADNI: Alzheimer’s Disease Neuroimaging Initiative
ANN: artificial neural network
CAD: computer-aided diagnosis
HC: healthy control
MCI: mild cognitive impairment
MRI: magnetic resonance imaging
OASIS: Open Access Series of Imaging Studies
PCA: principal component analysis
PET: positron emission tomography
SVM: support vector machine
ViT: vision transformer
